# Process development in *Hansenula polymorpha *and *Arxula adeninivorans*, a re-assessment

**DOI:** 10.1186/1475-2859-8-22

**Published:** 2009-04-15

**Authors:** Christoph Stöckmann, Marco Scheidle, Barbara Dittrich, Armin Merckelbach, Grit Hehmann, Georg Melmer, Doris Klee, Jochen Büchs, Hyun Ah Kang, Gerd Gellissen

**Affiliations:** 1 Institute of Biochemical Engineering, RWTH Aachen University, Worringer Weg 1, 52074 Aachen, Germany; 2 Institute of Textile and Macromolecular Chemistry, RWTH Aachen University, Pauwelsstr. 8, 52074 Aachen, Germany; 3 PharmedArtis GmbH, Forckenbeckstr. 6, 52074 Aachen, Germany; 4 Department of Life Sciences, Chung Ang University, 221 Heukseok-dong, Dongjak-gu, 156-756 Seoul, Korea

## Abstract

A range of industrial *H. polymorpha*-based processes exist, most of them for the production of pharmaceuticals. The established industrial processes lean on the use of promoters derived from *MOX *and *FMD*, genes of the methanol metabolism pathway. In *Hansenula polymorpha *these promoters are de-repressed upon depletion of a range of carbon sources like glucose and glycerol instead of being induced by methanol as reported for other methylotrophs. Due to these characteristics screening and fermentation modes have been defined for strains harbouring such expression control elements that lean on a limited supplementation of glycerol or glucose to a culture medium. For fermentation of *H. polymorpha *a synthetic minimal medium (SYN6) has been developed. No industrial processes have been developed so far based on *Arxula adeninivorans *and only a limited range of strong promoter elements exists, suitable for heterologous gene expression. SYN6 originally designed for *H. polymorpha *provided a suitable basis for the initial definition of fermentation conditions for this dimorphic yeast. Characteristics like osmo- and thermotolerance can be addressed for the definition of culture conditions.

## *Hansenula polymorpha *and *Arxula adeninivorans *and their competitive environment

In the last three decades a wide range of recombinant proteins, especially pharmaceuticals, have been produced based on heterologous gene expression in bacterial organisms, mammalian cells and several yeasts and fungi [1-3]. Production processes had to be developed that employ platforms which meet both, the demand for efficient mass production and criteria of safety and authenticity of the produced compounds. In this respect yeasts offer considerable advantages over alternative microbial and mammalian cell systems in providing low-cost screening and production systems for authentically processed and modified proteins. The organisms meet safety prerequisites in that they do not harbour pyrogens, pathogens or viral inclusions [4,5]. Recent engineering of yeast hosts with the capability to add humanized N-glycans of the intermediate mannose type [6] or even the complex type [7] provides the option to produce biopharmaceuticals with human protein modifications. The recognition of yeasts as attractive expression platforms for biopharmaceuticals is met by genome analysis of an increasing number of yeast species, among others that of *Saccharomyces cerevisiae *[8] and *Hansenula polymorpha *[9].

As a consequence some early examples of FDA-approved biopharmaceuticals like insulin [2] and hepatitis B vaccines [10,11] have been produced in the baker's yeast *S. cerevisiae*. However, certain limitations and drawbacks are encountered when using this system: *S. cerevisiae *tends to hyperglycosylate recombinant proteins; N-linked carbohydrate chains are terminated by mannose attached to the chain via an α1,3 bond, which is considered to be allergenic. The limited carbon source utilization imposes restrictions on the design of fermentation processes; due to the preferential use of episomal vectors instabilities of recombinant strains and as a result batch inconsistencies of production runs are of major concern [12].

Therefore an increasing number of alternative yeast systems have been defined that can potentially overcome the described limitations of the traditional baker's yeast. The availability of a wide-range yeast vector system (CoMed™) enables the assessment of several yeasts in parallel for their capability to produce a particular protein in desired quality with a single vector to identify an optimal host at the beginning of a product and process development. For expression control the wide-range vector contains a constitutive *TEF1 *promoter derived from various sources that is active in all yeast species analyzed so far. If needed this promoter element can easily be substituted during further strain development by a promoter optimal for the defined platform [[13], Additional file 1]. Out of the plethora of addressable species we describe in this article methylotrophic *H. polymorpha*, a recognized producer of biopharmaceuticals and other recombinant proteins, and dimorphic *Arxula adeninivorans*, a novel platform that has yet to establish itself for industrial applications. First experiments indicate that screening and fermentation conditions based on minimal SYN6 medium (SYN6) with glucose supplementation as described in this article can also be applied to yeast platforms others than *H. polymorpha *and *A. adeninivorans*.

**Figure 1:**
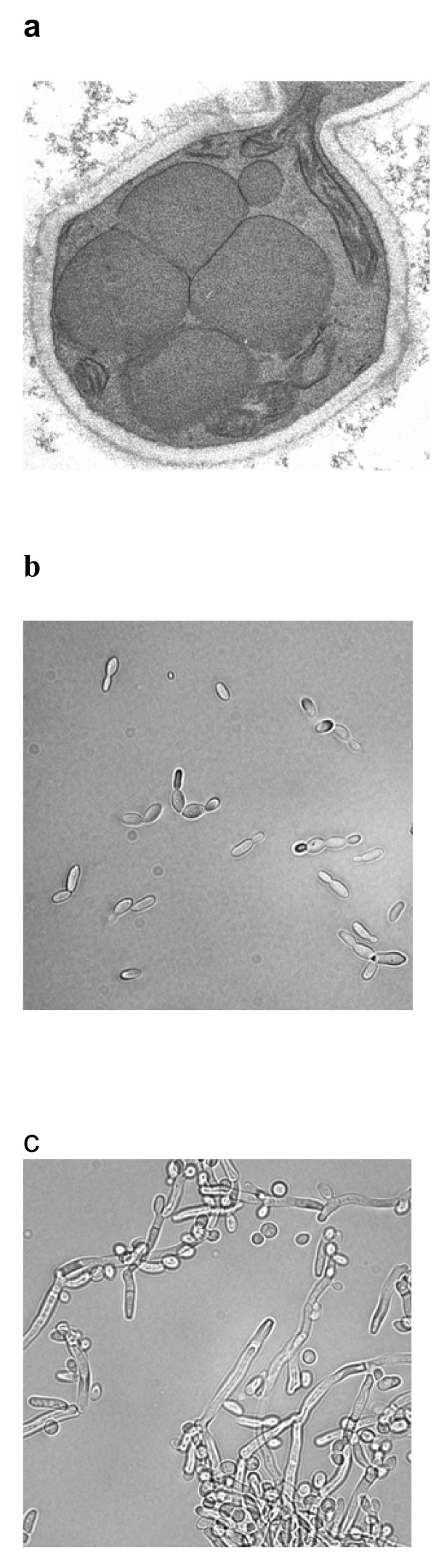
**Microscopy analysis of *Hansenula polymorpha *and *Arxula adeninivorans *cells**. a *H. polymorpha *cell grown in a methanol-supplemented chemostat (courtesy by M. Veenhuis, Groningen). Under these growth conditions proliferation of peroxisomes is induced. b *A. adeninivorans *cells grown at 37°C. c *A. adeninivorans *cells grown at 42°C. Dimorphic *A. adeninivorans *grows in filamentous forms above 42°C.

*H. polymorpha *(*Pichia angusta*) belongs to a limited number of yeast species that are able to utilize methanol as a sole energy and carbon source. Two out of three basic strains with unclear relationships, different features, and independent origins are biotechnologically applied: strain CBS4732 (CCY38-22-2, ATCC34438, NRRL-Y-5445) and DL-1 (NRRL-Y-7560, ATCC26012) and auxotrophic derivatives thereof [14,15]. A micrograph of a *H. polymorpha *cell prepared from a chemostat with a methanol feed is shown in Fig. 1a.

The range of biotechnologically applied methylotrophic yeasts furthermore includes *Candida boidinii*, *Pichia methanolica*, and *Pichia pastoris *[16]. In all instances most examples of heterologous gene expression are linked to strong and adjustable promoters derived from genes of the methanol utilization pathway [12,17], most commonly the elements derived from the alcohol oxidase genes, namely *AOX1 *from *P. pastoris *[16-18], *MOX *from *H. polymorpha *[14,17]; *AOD1 *from *C. boidinii *[16,17,19] and *AUG1 *(now designated *MOD1*) from *P. methanolica *[16,17,20]. In *H. polymorpha *the *FMD *(formate dehydrogenase) promoter, derived from another methanol utilization pathway gene of similar regulation, has found preferential application to established industrial processes [14,16].

*A. adeninivorans *(*Blastobotrys adeninivorans*) is a yeast with unusual characteristics. It is a dimorphic species and can utilize adenine, xanthine, uric acid, putrescine and *n*-alkylamines as carbon, nitrogen or energy sources in addition to glucose. Like *H. polymorpha *it is a nitrate-assimilating, thermo- and osmotolerant organism. A distinctive feature is a temperature-dependent dimorphism with mycelial structures formed at temperatures above 42°C [5,12] [Fig. 1b, c]. For Fe(II)-oxidase Afet3p, O-glycosylation was observed to be restricted to the budding cell status [21]. It remains to be shown whether this differential O-glycosylation pattern in correlation to the morphological status is also present in recombinant and other host proteins. Again, several strains have been identified after its first description as *Trichosporon adeninovorans *[22]. Most of the research and the biotechnological applications have been performed with strain LS3 (PAR-4), isolated in Sibiria by Kapultsevich, and a range of auxotrophic mutants have been generated [5,12]. Strain 135 is a mutant that forms mycelial structures at 30°C [23]. Recently auxotrophic host strains for heterologous gene expression have been generated based on strains CBS7350 and CBS1738 (see Tab. 1). So far, no industrial *A. adeninivorans*-based process exists. For expression and fermentation studies on a laboratory scale heterologous genes were mostly expressed under control of *TEF1*, a constitutive *A. adeninivorans*-derived promoter of appropriate strength [5].

For description we selected established *H. polymorpha*-based processes with strains expressing a heterologous gene under control of the adjustable *FMD *and *MOX *promoters and *A. adeninivorans*-based lab scale processes with strains expressing a heterologous gene under control of the constitutive *A. adeninivorans*-derived *TEF1 *promoter, with culturing conditions that can possibly be applied to the assessment of other yeasts with constitutive heterologous gene expression. Micrographs of the two selected platforms are shown in Fig. 1.

## *H. polymorpha*-based processes under control of *MOX *and *FMD *promoters – a re-assessment

*MOX *and *FMD *are genes encoding enzymes of the methanol utilization pathway that is shared by all methylotrophic yeasts. The enzyme components of this pathway and their control have been reviewed extensively in the recent past [4,17,19]. The genes of this pathway are described to be tightly regulated; they are highly repressed in the presence of non-limiting concentrations of glucose and strongly induced if methanol is used as a carbon source [17]. Methylotrophic growth is furthermore accompanied by a massive proliferation of peroxisomes in which several methanol-metabolizing enzymes are compartmentalized [19,24]. However, it soon became evident that activation of methanol pathway promoters did not depend on the presence of methanol in *H. polymorpha *in contrast to the situation in the other methylotrophs [16]. For all other methylotrophic yeast species an inductive activation of such promoters has been stated that is strictly dependent on the presence of methanol [17]. As a consequence several *H. polymorpha*-based industrial fermentation processes have been defined that lean on glucose or glycerol supplementation in suitable concentrations to a culture broth without any methanol additions [1,25,26].

The distinct feature of the *H. polymorpha*-derived methanol pathway promoters was elucidated, when new tools of genomics and postgenomic analysis became available. After sequencing the entire genome of strain CBS4732 [9] a cDNA microarray was constructed that allowed comprehensive gene expression profiling [27,28]. When analyzing the transcriptome of *H. polymorpha *strains of glucose-supplemented growth and after transition to methanol-supplemented growth it became evident that the methanol dissimilation genes including *MOX *and *FMD *are activated by de-repression upon carbon source limitation and depletion and not upon induction by methanol. In contrast genes of peroxisome biogenesis and proliferation are induced by methanol [14,15].

With respect to these findings, recombinant *H. polymorpha *strains expressing a GFP reporter gene under control of the *FMD *promoter were screened applying glucose- or glycerol-supplemented media to strain culturing.

**Figure 2:**
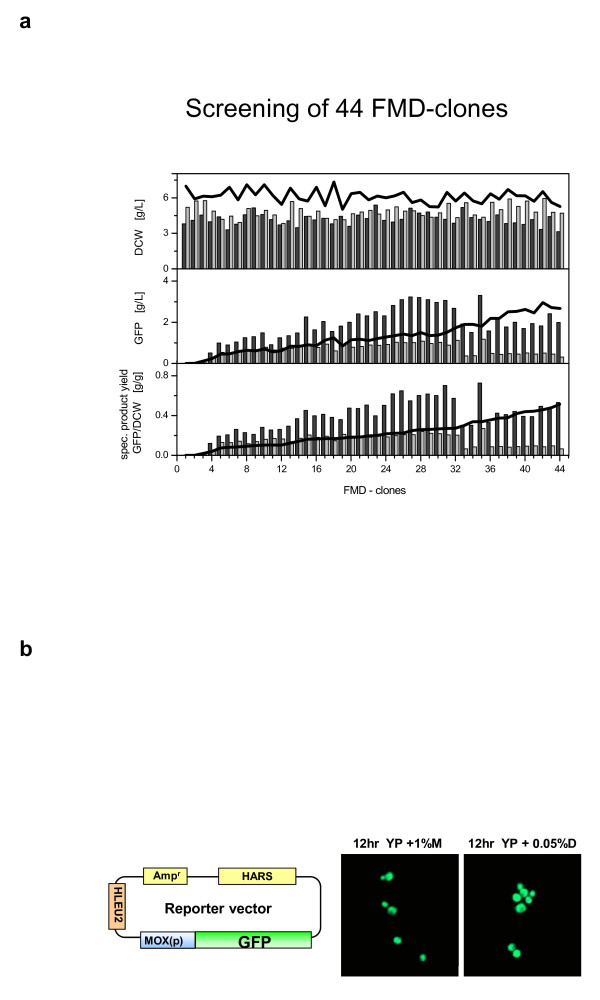
**Methanol pathway promoter-controlled GFP production in recombinant *H. polymorpha *strains**. A. Screening of CBS4732-derived transformants producing GFP under control of the *FMD *promoter. Screening of 44 different *H. polymorpha *pC10-FMD (P_FMD_-GFP) clones cultured under different cultivation modes; screening experiments were performed in deep-well-plates for batch and in FeedBead96-plates for fed-batch cultivation. Gas permeable sealings were used as sterile closures. To reduce the evaporation during the cultivation, water-saturated air was used for gassing. To compare the results from the different cultivation modes an identical carbon source concentration of 16.63 g/L were applied to each fermentation (batch with glycerol, batch with glucose, fed-batch with glucose). The batch cultivations were stopped when the stationary growth phase (glycerol 21 h, glucose 16 h) was reached and the fed-batch cultivations were stopped when 16.63 g/L glucose were released from the FeadBeat96-plate (16 h). Dry cell weight (DCW) was calculated from optical density measurements in a PowerWave ×340 micro titer plate reader. Green Fluorescent Protein (GFP) was measured at 485 nm excitation and 520 nm emission wavelengths. Cultivation conditions: shaking frequency 400 rpm, shaking diameter 50 mm, temperature 37°C, filling volumes 300 μL per well; media: SYN6-MES with 16.63 g/L of different carbon sources. Carbon sources: batch mode with glycerol (black line), batch mode with glucose (grey bars), fed-batch mode with glucose (black bars); inocula ratio 1:30. B. GFP production in a DL-1-derived transformant under control of the *MOX *promoter. *H. polymorpha *transformants producing GFP under control of *MOX *promoter were cultured for 12 h on YP medium containing 10 g/L methanol (YP+1%M) or 0.5 g/L glucose (YP+0.05%D) and then analyzed by confocal microscopy.

High throughput screening experiments are usually performed in a batch-mode [29]. Jeude *et al*. described a system for the slow release of glucose from a silicone elastomere matrix in shake flask [30]. They showed the advantage of using fed-batch cultivations in contrast to batch cultivations in small scale, where up to 420-fold increased GFP production was reached [30]. The slow-release technique was transferred to a deep-well-plate format. In these plates the glucose-containing silicone matrix is fixed at the bottom of each well. Hence, 96 parallel fed-batch-cultivations can be performed. A comparative screening with different *H. polymorpha *pC10-FMD (P_FMD_-GFP) strains (strains of a CBS4732 background) [31] was performed in batch mode with glycerol and glucose supplementation and in fed-batch mode with glucose supplementation [Fig. 2a]. The comparison attests that clones with an *FMD *promoter-controlled gene expression can be screened in small volumes of media supplemented with glycerol or glucose. This supports that methanol pathway promoters are activated upon glycerol and glucose depletion as suggested by the transcriptome analysis. Interestingly some clones of maximal productivity identified under conditions of glucose starvation (fed-batch conditions) are not identical to those identified under condition of glycerol starvation (batch conditions). The reasons for this phenomenon remain obscure. *MOX *promoter-driven GFP expression under glucose starvation conditions was corroborated for recombinant strains of a DL-1 background cultured in a different medium and on a larger scale as shown by fluorescence analysis in Fig. 2b.

**Figure 3:**
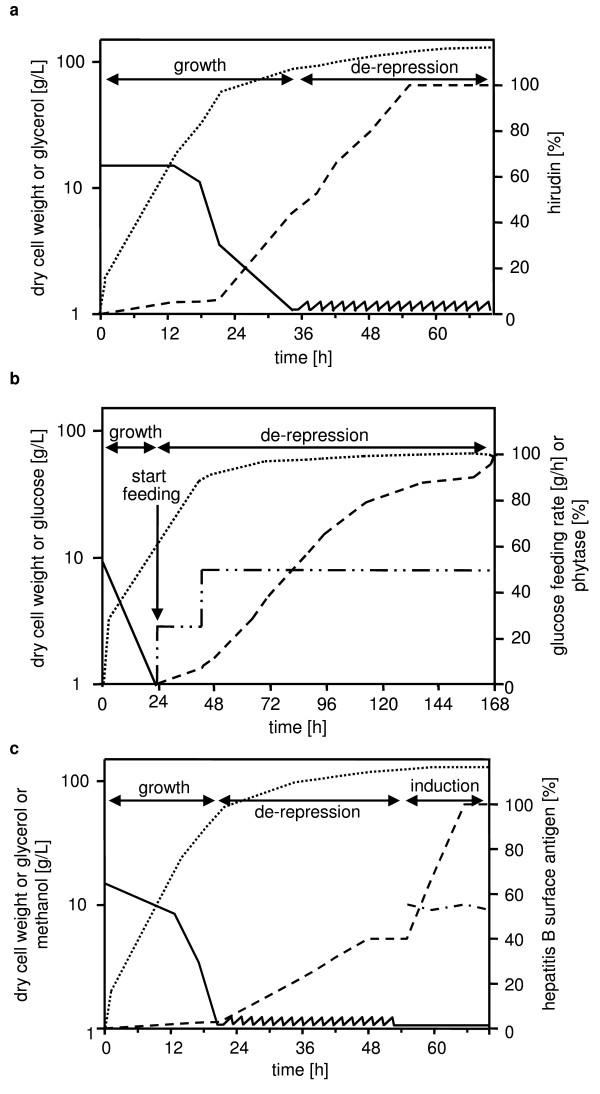
**A, B, C: Schematic depiction of *H. polymorpha*-based fermentation processes**. The figures are modified versions of figures from previous publications. For further details see text. a fermentation of a hirudin-producing strain (*MOX *promoter-controlled expression, glycerol starvation), b fermentation of a phytase-producing strain (*FMD *promoter-controlled gene expression, glucose starvation), c fermentation of a HBsAg-producing strain (*FMD *promoter-controlled expression, glycerol starvation, followed by a glycerol/methanol feed). dotted line: dry cell weight, solid line: glycerol (a, c) or glucose (b), chain dotted line: glucose feeding rate (b) or methanol (c), dashed line: product of a, b and c respectively.

The commercial success of a recombinant product does not depend only on the characteristics of the microbial host but also to a large extent on the definition of efficient fermentation processes. In case of *H. polymorpha*, a defined minimal mineral medium has been developed designated SYN6 [25,32]. It is composed of salts, vitamins and trace elements to support growth to high cell densities and it has to be adjusted to appropriate pH conditions and has to be supplemented with suitable carbon sources (for details of components see [32]). The fermentation strategy in previous established process developments with *FMD *or *MOX *promoter-driven production relied on growth using either glucose or glycerol in the beginning of fermentation, followed by carbon source limitations in a second phase. The respective fermentation parameters for the supplementation of glucose or glycerol have been defined prior to the elucidation of the transcriptome profile described before. In a single process example the fermentation broth was supplemented in a late phase with methanol. The three different fermentation modes are schematically depicted in Fig. 3a–c.

An early example for a glycerol starvation process is the production process for hirudin [33-35] [Fig. 3a]. As in all other described processes expression vectors were constructed to transform the uracil-auxotrophic strain RB11, like strain MedHp1 (Tab. 1) a derivative of strain LR9 [36-38]. The vectors contain an expression cassette with a hirudin sequence under control of the *MOX *promoter. For secretion it was fused to a secretion leader, derived from the pheromone precursor MFα1p [33]. Fermentation on a 35 liter scale was carried out at 30°C in SYN6 at pH 5. It was started with 30 g/L of glycerol. After consumption of the carbon source after 35 hours a feed was initiated that added glycerol by a pO_2_-controlled feeding device. Hirudin production started after some 20 hours when the *MOX*-promoter was activated by de-repression under glycerol limitation. The subsequent feeding conditions supported the de-repressed status of the promoter by maintaining glycerol concentrations between 0.5 and 3.0 g/L. These feeding conditions resulted in an increasing accumulation of the product in the medium [33,34,39] [Fig. 3a].

Similar fermentation conditions were applied to culturing of production strains for the anticoagulant saratin [35,39] or for aprotinin [37], now applying the *FMD *promoter to expression control. This fermentation design was modified when developing a production process for the cytokine IFNalpha-2a [40,41]. IFNalpha-2a forms a disulfide bond between amino acids Cys1 and Cys98. Bond formation of the first amino acid Cys1 of the mature sequence provides a steric hindrance for correct maturation when processed from an MFα1/IFNalpha-2a precursor. Accordingly a large share of secreted recombinant hirudin consisted of incorrectly processed molecules with N-terminal extensions. This could be overcome by co-production of the processing enzyme Kex2p, however at the expense of a more pronounced proteolytic degradation. To minimize this degradation pH was lowered from pH 5 (as applied to standard fermentations) to pH 2–3. The glycerol starvation conditions for *FMD *promoter de-repression remained unchanged.

For the production of phytase an extremely efficient production process has been developed. In this process all steps and components of the process followed a rationale of efficiency and cost-effectiveness. This rationale provoked an assessment of glucose as sole carbon source for fermentation [42]. In a fermentation of a phytase production strain with *FMD*-controlled expression on 2000 L scale glucose was supplemented as 20 g/L. Upon depletion, a glucose-limiting feed was initiated that added the carbon source with a stepwise increasing feeding rate in correlation to the cell mass. In this glucose starvation process a final yield of 13.5 g/L phytase was observed [42,43] [Fig. 3b].

The only established industrial fermentation process with methanol supplementation is that for the production of the hepatitis B surface antigen HBsAg, the first biopharmaceutical produced in *H. polymorpha*. Several processes for this vaccine have been described that are based on both, *MOX *or *FMD*-controlled expression [11]. In Fig. 3c a typical fermentation process is schematically shown. The batch phase and a first fed-batch phase is similar to the examples of glycerol starvation described before [Fig. 3a]. In contrast a mixture of glycerol and methanol is fed during the last hours of fermentation. Obviously this results in an inductive increase of HBsAg production. However, in view of the transcriptome profiling it seems that methanol supplementation does not induce the *FMD*-controlled expression of the heterologous antigen gene, but it induces membrane proliferation. As the HBsAg is produced as particles with the recombinant antigen inserted into host-derived membranes, methanol is considered to provide a balanced co-production of both particle components in high titers [10,11].

The selected process examples demonstrate the possibility to develop efficient screening and fermentation processes for strains with *MOX*- or *FMD*-driven heterologous gene expression without methanol supplementation to a medium.

## Culturing of *Arxula adeninivorans *strains

For *A. adeninivorans *industrial process parameters have not been defined yet and mainly cultivations on a shake flask scale have been carried out so far. Most of the current expression studies are based on wild type strain LS3 [44] or its leucine-auxotrophic derivative G1211. Additional strains and leucine-auxotrophic mutants thereof have been established more recently (see Tab. 1).

Acid phosphatase production was characterized in fermentations of both strain LS3 and a recombinant strain expressing the *APHO1 *gene under control of the strong *TEF1 *promoter [45]. Using the Plackett-Burman design three variables (pH, sucrose concentrations, and peptone concentration) were optimized for medium composition, a roughly four times enhancement was observed in media containing 39 g/L sucrose and 16 g/L peptone at pH 3.8 [46].

Shake flask cultures of *A. adeninivorans *strains were analyzed using a device for online measurement of the respiration rates (RAMOS, respiratory activity monitoring system) [47,48]. This device had previously been applied to the analysis of *H. polymorpha *cultures and to alternative platforms [49-52].

**Figure 4:**
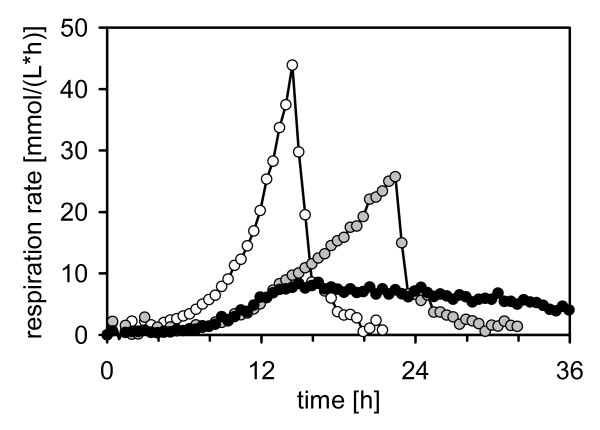
**Assessment of minimal media for *Arxula adeninivorans *LS3 by measuring respiration rates in shake flask cultures (for operating conditions and media formulation see **[25]**)**. Original YMM (black circles), containing poor concentrations of calcium (0.338 mg/L) and iron (0.041 mg/L), led to poor culture respiration rates below 10 mmol/(L*h). Modified YMM* (grey circles) is pH-buffered with MES and contains higher concentrations of calcium (272.8 mg/L) and iron (2.1 mg/L), thus leading to a more vital cultivation as shown by the higher respiration rate of up to 25 mmol/(L*h) and a condensed cultivation time of 24 hours (see drop of respiration, signalling the depletion of the carbon source glucose). SYN6-MES (open circles), containing exceeding nutrient quantities, provided non-limited growth of the culture as shown by the distinct exponential increase of respiration to 45 mmol/(L*h) and an early cease of respiration after only 15 hours, again signalling the depletion of carbon source.

In a first series yeast minimal medium (YMM) was assessed. Prior to any practical experiment, YMM ammonium concentration of the standard medium was raised from 2.2 mmol N/g glucose to 4.6 mmol N/g glucose since theoretical material balancing revealed a severe lack of nitrogen with regard to the average nitrogen content of yeast. The culture broth was additionally supplemented with calcium and iron in higher concentrations as well as with MES (2-[N-morpholino]ethanesulfonic acid) for buffering to result in YMM* [25]. Despite nitrogen addition to YMM, the respective cultures remained limited as shown representatively for *A. adeninivorans *LS3 in Fig. 4, black circles. Initially, culture respiration increased exponentially, but was then limited to 9 mmol/(L*h) after 13 hours, and continued to decline over fermentation time. Finally only ca. 6.6 g dry cell weight (DCW)/L was obtained.

In fermentations of strain LS3 in MES-buffered YMM* the respiration rate exponentially increased followed by a linear increase presumably indicating a nutrient deficiency. After 24 hours, respiration rate dropped upon glucose depletion. [Fig. 4, grey circles].

Subsequently glucose-supplemented SYN6 was assessed for applicability to shake flask cultures of *A. adeninivorans*. The high nutrient concentrations of standard SYN6 remained unchanged. Again, the pH of SYN6 had to be buffered with MES (SYN6-MES, see [25] for detailed description) for pH stabilization between 6.4 and 5.3. Fig. 4, open circles shows the respiration rate of a shake flask culture with strain LS3 in SYN6-MES. The described culture course adverts to the non-limited growth of *A. adeninivorans *in the respective shake flask culture. Thus, favourable non-limiting growth conditions for *A. adeninivorans *in shake flasks were proven to be developed.

Additionally suitable fed-batch conditions for high cell density fermentations (HCDF) in SYN6 were found. These conditions were assessed for culturing a recombinant *A. adeninivorans *strain producing phytase under control of the *TEF1 *promoter [5,53]. During the feeding under glucose-limitation phytase was secreted to maximal titres of ca. 900 FTU/mL (one FTU equates to the phytase amount liberating 1 μmol of inorganic phosphate per minute at pH 5.5 and 37°C) [25]. Thus, the growth conditions defined for shake flask cultures and HCDF of *A. adeninivorans *wild type strain LS3, proved to be applicable for the phytase-producing recombinant *A. adeninivorans *strain. Finally the phytase-producing strain was cultured under pressurized conditions in a 50 L STR, again using a SYN6-derived medium for culturing. During the fed-batch phase the reactor pressure was increased stepwise up to 5 bar. After 42 h cells had grown up to 224 g/L. Phytase amount increased up to 10 × 10^6 ^FTU. Fermentations under pressurized conditions may result in increased product yields and shorter fermentation time [54].

The conditions of culturing can potentially be applied to screening and culturing of other yeasts expressing a foreign gene under control of a constitutive *TEF1 *promoter – a key element of the CoMed™ system described in the following section.

## The CoMed™ system

**Figure 5:**
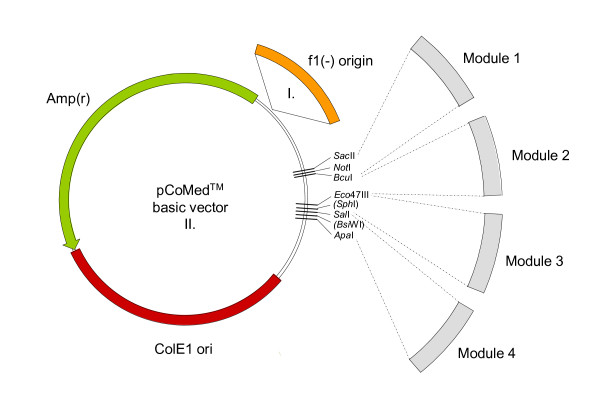
**CoMed™ vector system**. The basic vectors are derived from standard vectors with an engineered multiple cloning site (MCS) for the uptake of various modules. In its basic form all modules are functional in all yeasts tested so far. Module 1: *ARS/CEN *sequences from various sources (optional); Module 2: rDNA targeting sequences (NTS2-ETS-18SrDNA-ITS1 from various sources); Module 3: selection markers (i.e. *kanMX*, *hph*, *leu2, ura3 *and combinations thereof); Module 4: expression cassettes consisting of a *TEF1 *promoter from various sources – cloning site – terminator. The wide-range *TEF1 *promoter can easily be replaced by alternative strong species-specific promoters like *MOX *or *FMD*. For further details see text.

While all established expression systems are distinguished by certain favorable characteristics, it is evident that no single system is optimal for all proteins. An initial selection can result in costly time- and resource-consuming failures. It is thus advisable to assess several platform candidates in parallel for criteria such as productivity, appropriate processing and modification. Production of interleukin IL-6 in various yeast platforms has recently been described as a striking example for the necessity of a comparative evaluation of several yeasts. Correct processing from an MFα1/IL-6 precursor was observed in *A. adeninivorans *whereas N-terminally truncated molecules were secreted from *S. cerevisiae *and *H. polymorpha *hosts [55]. A novel yeast/vector system provides a versatile tool to address simultaneously with a single vector a range of yeasts like the two described before, namely *Hansenula polymorpha *and *Arxula adeninivorans*, and others like *Saccharomyces cerevisiae*, *Pichia pastoris*, *Kluyveromyces lactis*. In its basic form the vector is composed of genetic modules that are functional in all yeasts, namely an rDNA targeting sequence, an appropriate selection marker and an expression cassette under control of a *TEF1 *promoter from various sources. The CoMed™ system has recently been described and some application examples have been provided [13,55-57]. The basic design of the vector and a selection of addressable yeast species are shown in Fig. 5 and Additional file 1.

It is desirable that the range of yeasts addressed in parallel can be also assessed in parallel for optimal performance in a given case. First experiments indicate that SYN6 and derivatives thereof are suitable minimal media for yeasts others than *A. adeninivorans *and *H. polymorpha*. The general use of the constitutive *TEF1 *promoter is expected to ensure screening and fermentation conditions similar to those described for *H. polymorpha *and *A. adeninivorans*.

## Competing interests

The authors declare that they have no competing interests.

## Authors' contributions

CS, MS, BD, DK and JB contributed to the reviewed and new data on screening under glucose limitations and on the fermentation design for *Arxula adeninivorans *cultures, HK is the principal scientist for the *Hansenula polymorpha *microarray and she contributed the data on GFP-production in DL-1 under glucose fermentation. AM, GH, GM and GG performed the work on the CoMed system. GG was a project partner in the microarray work and participated in the projects and the publications on *Hansenula polymorpha *reviewed in this manuscript. All authors read and approved the manuscript.

## Supplementary Material

Additional file 1 **Table 1 Components of the CoMed™ system**. The table contains a selection of genetic components and yeast strains of the CoMed strain/vector system. Click here for file
