# Supplementary material for: Process development in Hansenula polymorpha and Arxula adeninivorans, a re-assessment
Source: Microb Cell Fact. 2009 Apr 15;8:22. doi: 10.1186/1475-2859-8-22 (PMC2676251; doi:10.1186/1475-2859-8-22)
Supplement: Additional file 1 — Table 1 Components of the CoMed™ system. The table contains a selection of genetic components and yeast strains of the CoMed strain/vector system. [file 1475-2859-8-22-S1.doc]

**Table 1. Components of the CoMed^TM^ system (selection)**

**___________________________________________________________________**

**Vector components**

**___________________________________________________________________**

**Module 1: *ARS/CEN* sequences**

*HARS1* (*H. polymorpha*-derived autonomously replication sequence)

*ARS* (*S. cerevisiae*)

*CEN* (*S. cerevisiae*)

**_________________________________________________________________________________**

**Module 2: rDNA targeting sequences**

*NTS2-ETS-18SrDNA-ITS1* (*H. polymorpha*, *Arxula adeninivorans*)

Others

_________________________________________________________________________________

**Module 3: selection marker**

1. **dominant**

*TEF1* promoter (*A. gossypii, A. adeninivorans) - hph* (*E.coli*) - *TEF* terminator (hygromycin resistance)

*TEF1* promoter (*A. gossypii, A. adeninivorans*) - *kanMX* (*E. coli*) - *TEF* terminator (gentamycin resistance)

1. **complementation**

*URA3* (*S. cerevisiae*)

*LEU2* (*S. cerevisiae*, *A. adeninivorans*)

*dLEU2* (*A. adeninivorans*) (deficient promoter)

*TRP1* (*S. cerevisiae*)

**_________________________________________________________________________________**

**Module 4: expression cassettes consisting of promoter - cloning site – terminator**

**(terminator mostly from *MOX* but also from *TEF* and *PHO5*)**

**wide-range**

*TEF1* promoter (*A. adeninivorans*, *A. gossypii*)

**restricted range**

*NR* promoter (*H. polymorpha*) (nitrate-assimilating species)

*TPS1* promoter (*H. polymorpha*) (trehalose-accumulating species)

**Species-specific for *H. polymorpha***

*MOX*

**_________________________________________________________________________________**

**___________________________________________________________________**

Yeast strains (selection)

**___________________________________________________________________**

**Species auxotrophies**

***Arxula adeninivorans* (Sibirian isolate) wild type, *leu2***

***Arxula adeninivorans* (CBS7350) wild type, *leu2***

***Arxula adeninivorans* (CBS1738) wild type, *leu2***

***Hansenula polymorpha* (CBS4732) wild type, *ura3*, *leu2 ura3, arg1 leu2 ura,***

***ade1 leu2 ura3***

***Kluyveromyces lactis met^-^ ura3***

***Pichia pastoris* wild type, *ura3, ura3 his3***

***Saccharomyces cerevisiae* wild type, *ura3*, *leu2 ura3 trp1 lys2***

***Yarrowia lipolytica E150* wild type, *ura3, leu2, ura3 leu2***

**_________________________________________________________________________**
